# The Importance of Pan‐Immune‐Inflammation Value Score in Locally Advanced Rectal Cancer

**DOI:** 10.1002/iid3.70227

**Published:** 2025-07-24

**Authors:** Mahmut Uçar, Mukaddes Yılmaz, Eda Erdiş, Birsen Yücel

**Affiliations:** ^1^ Department of Medical Oncology, Faculty of Medicine Sivas Cumhuriyet University Sivas Turkey; ^2^ Department of Radiation Oncology, Faculty of Medicine Sivas Cumhuriyet University Sivas Turkey

**Keywords:** PIV, prognosis, rectal cancer, survival

## Abstract

**Aim:**

In this study, we aimed to observe the prognostic significance of the pan‐immune‐inflammation value (PIV) score calculated at the time of diagnosis in patients with locally advanced rectal cancer, as well as its effect on treatment response, survival, and prognosis.

**Material and Method:**

This retrospective, single‐center observational study was designed to analyze patients with nonmetastatic (stages II–III) rectal cancer who received neoadjuvant treatment, categorized into two groups: PIV‐L (*n* = 67, 50%) and PIV‐H (*n* = 67, 50%). The median PIV score was used for cutoff determination. Survival analysis was applied. Univariate and multivariate Cox regression analyses were used to determine prognostic factors.

**Results:**

Preoperative clinical lymph node status (*p* = 0.011), liver metastasis (*p* = 0.028), carcinoembryonic antigen (CEA; *p* = 0.013), and cancer antigen 19.9 (CA19.9; *p* = 0.040) levels; pathological complete response (*p* = 0.035); tumor regression score (*p* = 0.030); postoperative lymph node status (*p* = 0.019); tumor deposits (*p* = 0.035); and budding (*p* = 0.043) were statistically different between the groups. The 5‐ and 10‐year overall survival (OS) rates were 77% versus 69% and 62% versus 38% in the PIV‐L and PIV‐H groups, respectively (*p* = 0.032). While the PIV score was prognostic for OS in univariate analysis (HR: 1.85, 95% CI: 1.04–3.31, *p* = 0.035), a result of insignificance was obtained in multivariate analysis (HR: 1.76, 95% CI: 0.98–3.01 *p* = 0.056). The 5‐ and 10‐year disease‐free survival (DFS) rates were 67% versus 54% and 56% versus 39% in the PIV‐L and PIV‐H groups, respectively, with the PIV‐H group showing a statistically significantly lower rate (*p* = 0.048). For DFS, the PIV score was found to be a statistically insignificant prognostic factor in univariate analysis (HR: 0.052, 95% CI: 0.99–2.86, *p* = 0.052) and recognized as an independent prognostic factor in multivariate analysis (HR: 1.87, 95% CI: 1.08–3.26, *p* = 0.026).

**Conclusion:**

A higher pretreatment PIV score was associated with poorer clinicopathological features, a worse treatment response, lower survival rates, and a poor prognosis for DFS.

## Graphical abstract

PIV score is a prognostic marker for rectal cancer. It can be used in the selection of high‐risk patients.

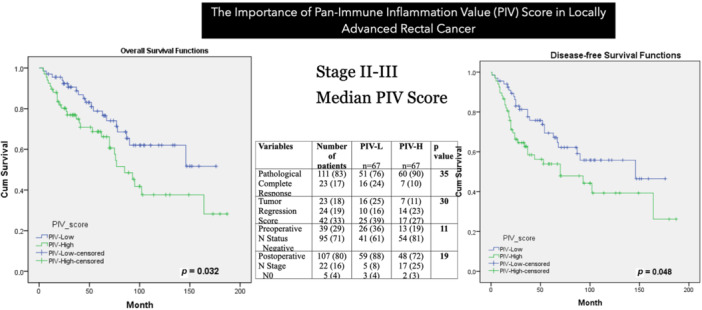

## 1 Introduction

Among all types of cancer, colorectal cancer is the third most commonly diagnosed and the second leading cause of death [1]. Between 5% and 10% of patients with rectal cancer present at a locally advanced stage [2]. Patients with advanced rectal cancer receive multimodal therapies, including radiotherapy, chemotherapy, and surgery [2, 3]. For stages II–III disease, the standard neoadjuvant treatment has consistently involved either long‐course chemoradiotherapy or short‐course radiotherapy, followed by surgery [4, 5]. Lately, there has been a shift toward using full‐dose chemotherapy and chemoradiotherapy before surgery for locally advanced rectal cancer, known as total neoadjuvant treatment (TNT) [6]. The treatment decision for these patients is typically based on radiological findings [7]. However, the reliability of staging through radiological analyses can be limited, resulting in potential overtreatment or undertreatment. Thus, in addition to radiological findings, there remains a need for additional evidence of tumor behavior. Individualized treatment planning considering patient and disease characteristics may enhance treatment success.

Cancer, a multifactorial disease, may be aggravated by chronic inflammation from various triggers, potentially contributing to about a quarter of cases [8]. Even though it plays a vital role in preventing cancer, uncontrolled inflammation may lead to alterations in the genetic makeup through DNA damage caused by various cytokines and chemokines, ultimately causing cancer development [9]. The relationship between systemic inflammation status and cancer may predict clinical outcomes in patients. Various investigations have revealed that changes in immune system components like neutrophils, monocytes, platelets, and lymphocytes, along with their equilibrium, may play a crucial role in determining the prognosis of cancer patients [10, 11]. There are many studies that show inflammation‐associated prognostic tools. Blood‐based biomarkers, such as serum calcium ion levels [12] and composite indices incorporating neutrophil percentage, LHb index, and monocyte count [13], have shown prognostic value. Moreover, the Onodera prognostic nutritional index, when combined with inflammatory markers, has proven useful in predicting postoperative complications such as anastomotic leakage in rectal cancer patients [14], highlighting the broader role of systemic inflammation in surgical outcomes. From a molecular perspective, bioinformatic studies have identified IL1RN and PRRX1 as immune‐related prognostic markers in colorectal cancer [15], while IL27RA has been recognized as a key immune modulator and therapeutic indicator in breast cancer based on single‐cell and transcriptomic analysis [16].

A new metric called pan‐immune‐inflammation value (PIV) has recently been created based on the counts of neutrophils, platelets, monocytes, and lymphocytes [17]. Including all inflammatory cells, this score has the potential to be a more reliable predictor of the tumor's inflammatory activity. Notably, previous studies have demonstrated that preoperative PIV is significantly associated with prognosis in nonmetastatic colorectal cancer and may be further refined when combined with the albumin‐to‐globulin ratio [18, 19]. Evidence from recent studies suggests PIV serves as a novel parameter indicative of the immune response in colorectal cancer, thereby emphasizing the clinical relevance of this immunological activity Limited evidence exists regarding the correlation between the PIV score and the prognosis and survival predictions in rectal cancer cases. The PIV could be vital in determining the treatment approach for locally advanced rectal tumors.

In this study, we aimed to observe the prognostic significance of the PIV score calculated at the time of diagnosis in patients with locally advanced rectal cancer, as well as its impact on treatment response, survival, and prognosis.

## 2 Patients and Methods

### 2.1 Study Design

This investigation was structured as a retrospective, observational study at a single center. The local ethics committee granted approval for the study protocol (Sivas Cumhuriyet University, Ethical Approval#: 2024/09‐48 on September 19, 2024). The research was conducted in line with the ethical guidelines outlined in the Declaration of Helsinki. Personal health data are recorded without violating the privacy or personal rights of the persons concerned and data are anonymised. Written consent was not necessary from participants due to the study's retrospective design and the protection of their anonymity.

### 2.2 Population and Sample

The study cohort comprised all rectal cancer patients who were monitored at the Oncology Center of the Faculty of Medicine at Sivas Cumhuriyet University in Turkey from 2007 to 2022. The research data were obtained from patients' medical records and the hospital information system. The study's inclusion criteria were being 18 years of age or older, having nonmetastatic (stages II–III) rectal cancer, and having received neoadjuvant treatment. On the other hand, the study's exclusion criteria included being younger than 18, having metastatic rectal cancer at admission, having a second primary cancer, and lacking adequate follow‐up data.

### 2.3 Data Collection

The study gathered and documented information on patients' characteristics such as age, comorbidities, Eastern Cooperative Oncology Group Performance Status (ECOG‐PS), laboratory results including neutrophil, monocyte, platelet, and lymphocyte counts, carcinoembryonic antigen (CEA), and cancer antigen 19.9 (CA19.9) levels, and pathological factors such as rectal cancer grade, invasion characteristics, TNM stages [20], and tumor localization using a predefined data collection tool.

PIV was calculated according to the equation PIV = neutrophil count × platelet count × monocyte count/lymphocyte count [17]. Since the area under the curve is statistically insignificant in the ROC analysis and the 95% confidence interval for this value includes 0.5, we used the median value as the cut‐off point [21]. Since survival analysis was performed in this study, the use of median PIV for the cut‐off value of PIV was preferred. A PIV score of 0 was described as below the median PIV, while a score of 1 indicated being at or above the median PIV. A PIV score of 0 was labeled as PIV‐low (PIV‐L), and a score of 1 was labeled as PIV‐high (PIV‐H).

### 2.4 Follow‐Up Procedure

All patients were followed up at 3–6 month intervals in the outpatient clinics of the oncology center. Recurrences, metastases, and types of metastases were recorded during the follow‐up visits. The time from the initiation of rectal cancer treatment to the first occurrence of rectal cancer recurrence, metastasis, or death was defined as disease‐free survival (DFS), whereas the time from rectal cancer diagnosis to death or the last follow‐up, regardless of recurrence or metastasis, was defined as overall survival (OS).

### 2.5 Statistical Analysis

Statistical analyses were conducted using IBM SPSS version 22 software. The power of the study was calculated by selecting an effect size of 0.5, group ratio of 1, with the G‐Power program (version 3.1.9.7). When *α* = 0.05, *β* = 0.10, 1 − *β* = 0.90, it was decided to include 134 individuals, and the power of the test was determined as 0.8914402. Descriptive statistics derived from the gathered data were presented as mean ± standard deviation or median with range for continuous variables and as frequencies and percentages for categorical variables. The normality of continuous variables was assessed using Shapiro–Wilk and Kolmogorov–Smirnov. For comparing categorical variables between groups, Pearson's *χ* ^2^ test and Fisher's exact test were employed. In comparing two independent groups, the independent samples *t*‐test was utilized for numerical variables conforming to normal distribution, and the Mann–Whitney *U* test for those not conforming. To determine the OS and DFS in patients with a PIV score of 0 and a PIV score of 1, Kaplan–Meier survival analysis was applied. Univariate and multivariate Cox regression analysis were used to determine prognostic factors. In this study, there was a small amount of missing data in some variables such as LVI, tumor deposits, grade, tumor regression grade, and pairwise deletion was applied for missing data. A probability (*p*) value of ≤ 0.05 was considered statistically significant.

## 3 Results

In this study investigating survival rates, the median value was used for cut‐off determination [22]. The median PIV value was determined to be 322 (range 68–2271). The study sample consisted of 134 consecutive nonmetastatic rectal cancer patients who were divided into two groups: PIV‐L (*n* = 67, 50%) and PIV‐H (*n* = 67, 50%). Most of the patients were male, with T4 disease predominating. In more than half of the patients, the tumor was distally localized. More than 70% of the patients received chemoradiotherapy as neoadjuvant treatment. There were statistically significant differences between the groups regarding preoperative clinical lymph node status (*p* = 0.011), CEA (*p* = 0.013), and CA19.9 (*p* = 0.040) levels. Demographic and clinical characteristics of PIV‐L and PIV‐H groups can be found in Table 1.

**Table 1:** Demographic and clinical characteristics of PIV‐L and PIV‐H groups.

Variables	Number of patients *n* = 134 (100%)	PIV‐L *n* = 67 (50%)	PIV‐H *n* = 67 (50%)	*p*
Sex				
Male	87 (65)	41 (61)	46 (69)	0.235
Female	47 (35)	26 (39)	21 (31)	
Comorbidity				
No	72 (54)	40 (60)	32 (48)	0.113
Yes	62 (46)	27 (40)	35 (52)	
ECOG PS				
0	57 (42)	30 (45)	27 (40)	0.600
1	69 (52)	32 (48)	37 (15)	
≥ 2	8 (6)	5 (7)	3 (5)	
Localization				
Proximal	11 (8)	6 (9)	5 (8)	0.946
Middle	49 (37)	24 (36)	25 (7)	
Distal	74 (55)	37 (55)	37 (55)	
Preoperative T stage				
T2	8 (6)	5 (8)	3 (5)	0.280
T3	30 (22)	19 (28)	11 (16)	
T4	93 (70)	42 (63)	51 (76)	
Tx	3 (2)	1 (1)	2 (3)	
Preoperative N status				
Negative	39 (29)	26 (36)	13 (19)	0.011
Positive	95 (71)	41 (61)	54 (81)	
Neoadjuvant therapy				
CRT	100 (75)	52 (78)	48 (72)	0.276
TNT	34 (25)	15 (22)	19 (28)	
Surgery type				
LAR	99 (74)	48 (31)	51 (76)	0.839
APR	35 (26)	19 (29)	16 (24)	
Adjuvant therapy				
No	47 (35)	21 (31)	26 (39)	0.235
Yes	87 (65)	46 (69)	41 (61)	
Recurrence pattern				
Local	10 (7)	5 (7)	5 (7)	0.628
Distant	24 (18)	8 (12)	16 (24)	0.057
Liver	5 (4)	—	5 (8)	0.028
CEA				
Normal	56 (45)	34 (56)	22 (34)	0.013
High	69 (55)	27 (44)	42 (66)	
CA19.9				
Normal	110 (89)	58 (95)	52 (84)	0.040
High	13 (11)	3 (5)	10 (16)	

*Note:* CEA normal: < 2.5 ng/mL, CA19.9 normal: < 37 U/mL.

Abbreviations: APR, abdominoperineal resection; CA19.9, cancer antigen 19.9; CEA, carcinoembryonic antigen; CRT, chemoradiotherapy; ECOG PS, Eastern Cooperative Oncology Group Performance Status; LAR, low anterior resection; PIV, pan‐immune‐inflammation value; TNT, total neoadjuvant treatment.

When examining the postoperative pathological characteristics of the groups, significant differences were found between them in terms of pathological complete response (*p* = 0.035), tumor regression score (*p* = 0.030), postoperative lymph node status (*p* = 0.019), tumor deposits (*p* = 0.035), budding (*p* = 0.043), and liver metastasis (*p* = 0.028). In contrast, postoperative T stage (*p* = 0.095), grade (*p* = 0.155), perineural invasion (*p* = 0.455), lymphovascular invasion (*p* = 0.270), and extracapsular invasion (*p* = 0.088) were similar. Table 2 presents postoperative histopathological features and survival of PIV‐L and PIV‐H groups.

**Table 2:** Postoperative histopathological features and survival of PIV‐L and PIV‐H groups.

Variables	Number of patients *n* = 134 (100%)	PIV‐L *n* = 67 (50%)	PIV‐H *n* = 67 (50%)	*p*
Pathological complete response				
No	111 (83)	51 (76)	60 (90)	0.035
Yes	23 (17)	16 (24)	7 (10)	
Tumor regression score				
Grade 0	23 (18)	16 (25)	7 (11)	0.030
Grade 1	24 (19)	10 (16)	14 (23)	
Grade 2	42 (33)	25 (39)	17 (27)	
Grade 3	37 (29)	13 (20)	24 (39)	
Postoperative T stage				
T0	23 (17)	16 (24)	7 (10)	0.095
T1	12 (9)	7 (10)	5 (8)	
T2	33 (25)	14 (21)	19 (29)	
T3	51 (38)	26 (39)	25 (37)	
T4	15 (11)	4 (6)	11 (16)	
Postoperative N stage				
N0	107 (80)	59 (88)	48 (72)	0.019
N1	22 (16)	5 (8)	17 (25)	
N2	5 (4)	3 (4)	2 (3)	
Grade				
Grade 1	39 (36)	21 (40)	18 (31)	0.155
Grade 2	63 (58)	30 (58)	33 (58)	
Grade 3	7 (6)	1 (2)	6 (10)	
Perineural invasion				
No	103 (80)	53 (82)	50 (79)	0.455
Yes	25 (20)	12 (18)	13 (21)	
Lymphovascular invasion				
No	113 (88)	59 (91)	54 (86)	0.270
Yes	15 (12)	6 (9)	9 (14)	
Tumor deposits				
No	77 (89)	43 (96)	34 (81)	0.035
Yes	10 (11)	2 (4)	8 (19)	
Tumor budding				
No	107 (86)	59 (92)	48 (80)	0.043
Yes	17 (14)	5 (8)	12 (20)	
Extracapsular invasion				
No	20 (74)	4 (50)	16 (84)	0.088
Yes	7 (26)	4 (50)	3 (16)	
Surgical margin				
Negative	118 (88)	61 (91)	57 (85)	0.213
Positive	16 (12)	6 (9)	10 (15)	
Overall survival				
The 5‐year (%)	74	77	69	0.032
The 10‐year (%)	50	62	38	
Disease‐free survival				
The 5‐year (%)	62	67	54	0.048
The 10‐year (%)	48	59	39	

Abbreviation: PIV, pan‐immune‐inflammation value.

**Figure 1:**
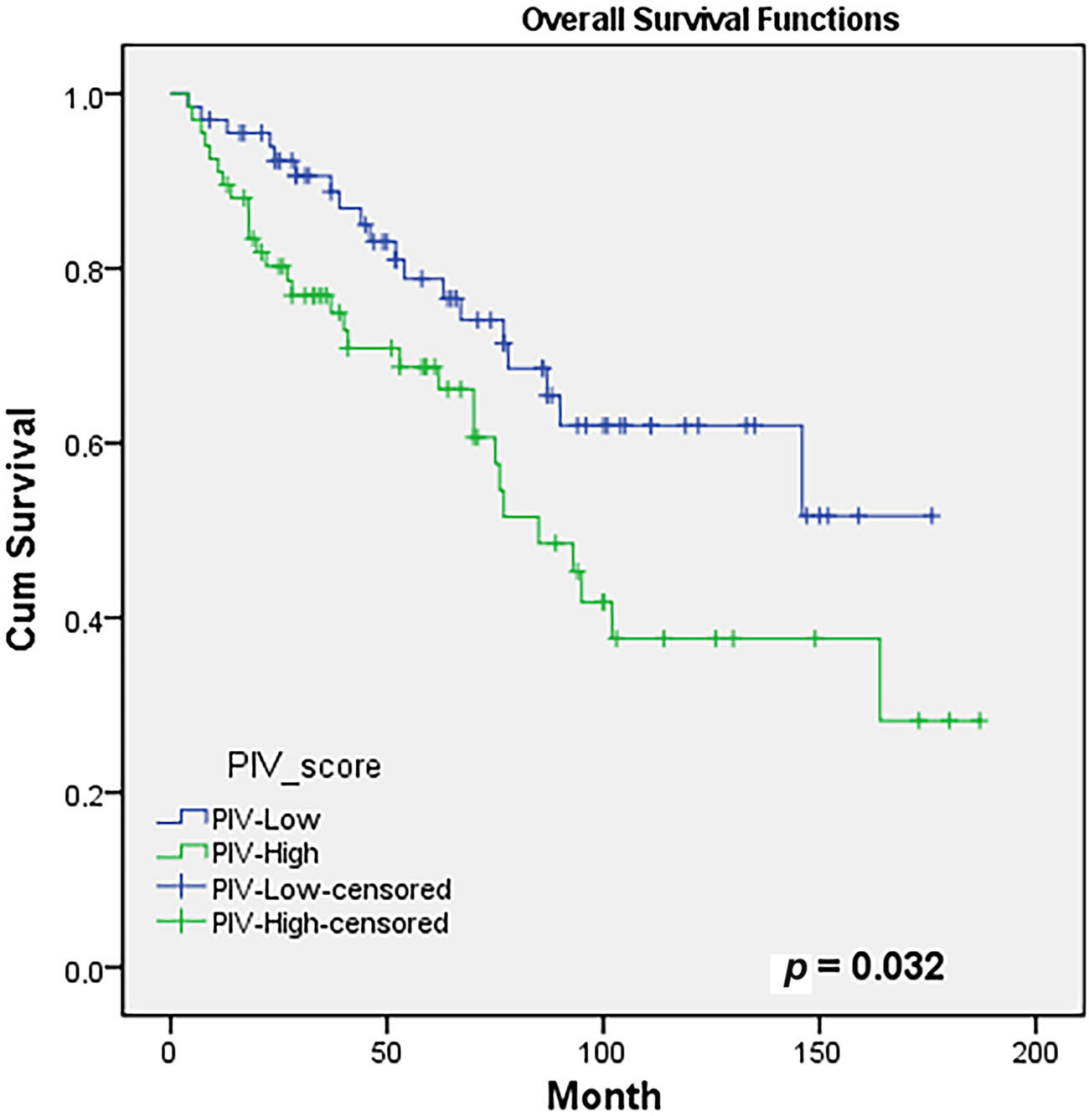
The Kaplan–Meier survival analysis with log‐rank test showing the overall survival outcomes of groups according to PIV‐H and PIV‐L statuses: overall survival curves of groups.

Evaluating the association between PIV score and OS utilizing the Kaplan–Meier test, OS rates at 5 years were 77% and 69%, and at 10 years, were 62% and 38% in the PIV‐L and PIV‐H groups, respectively (*p* = 0.032). Figure 1 presents the OS curves for the groups. In the univariate modeling assessing the factors affecting OS, the PIV score emerged as a prognostic factor (HR: 1.85, 95% CI: 1.04–3.31, *p* = 0.035), while a result statistical insignificance was identified in multivariate modeling (HR: 1.76, 95% CI: 0.98–3.01, *p* = 0.056). However, both univariate and multivariate analyses revealed that positive surgical margin and positive lymphovascular invasion were significant. Table 3 displays the prognostic factors affecting the OS of PIV‐L and PIV‐H groups.

**Table 3:** The prognostic factors affecting the overall survival of PIV‐L and PIV‐H groups.

Variables	Category	Univariate analysis		Multivariate analysis	
		HR (95% CI)	*p*	HR (95% CI)	*p*
PIV score	PIV‐L	RF		RF	
	PIV‐H	1.85 (1.04–3.31)	0.035	1.76 (0.98–3.01)	0.056
Sex	Male	RF			
	Female	1.58 (0.90–2.77)	0.109		
ECOG PS	ECOG 0	RF			
	ECOG 1	1.73 (0.93–3.21)	0.080		
	ECOG ≥ 2	2.16 (0.79–5.88)	0.132		
Localization	Proximal	RF			
	Middle	1.85 (0.42–8.01)	0.408		
	Distal	1.67 (0.39–7.02)	0.484		
Neoadjuvant treatment	CRT	RF			
	TNT	0.62 (0.34–1.13)	0.121		
Preoperative N stage	Negative	RF	RF	RF	
	Positive	2.07 (1.05–4.80)	0.034	2.06 (0.97‐4.35)	0.057
Surgery type	LAR	RF			
	APR	1.46 (0.81–2.62)	0.201		
Pathological complete response	No	RF			
	Yes	0.50 (0.19–1.26)	0.144		
Tumor regression grade	Grade 0	RF			
	Grade 1	1.47 (0.48–4.52)	0.500		
	Grade 2	1.82 (0.65–5.06)	0.252		
	Grade 3	2.89 (1.08–7.77)	0.035		
Surgical margin	Negative	RF		RF	
	Positive	2.57 (1.30–5.07)	0.006	2.41 (1.16–5.00)	0.018
Lymphovascular invasion	No	RF		RF	
	Yes	2.79 (1.37–5.66)	0.004	2.15 (1.01–4.55)	0.045
Adjuvant therapy	No	RF		RF	
	Yes	0.54 (0.30–0.97)	0.042	0.53 (0.27–1.00)	0.052

Abbreviations: APR, abdominoperineal resection; CI, confidence interval; CRT, chemoradiotherapy; ECOG PS, Eastern Cooperative Oncology Group Performance Status; HR, hazard ratio; LAR, low anterior resection; PIV, pan‐immune‐inflammation value; RF, reference; TNT, total neoadjuvant treatment.

**Figure 2:**
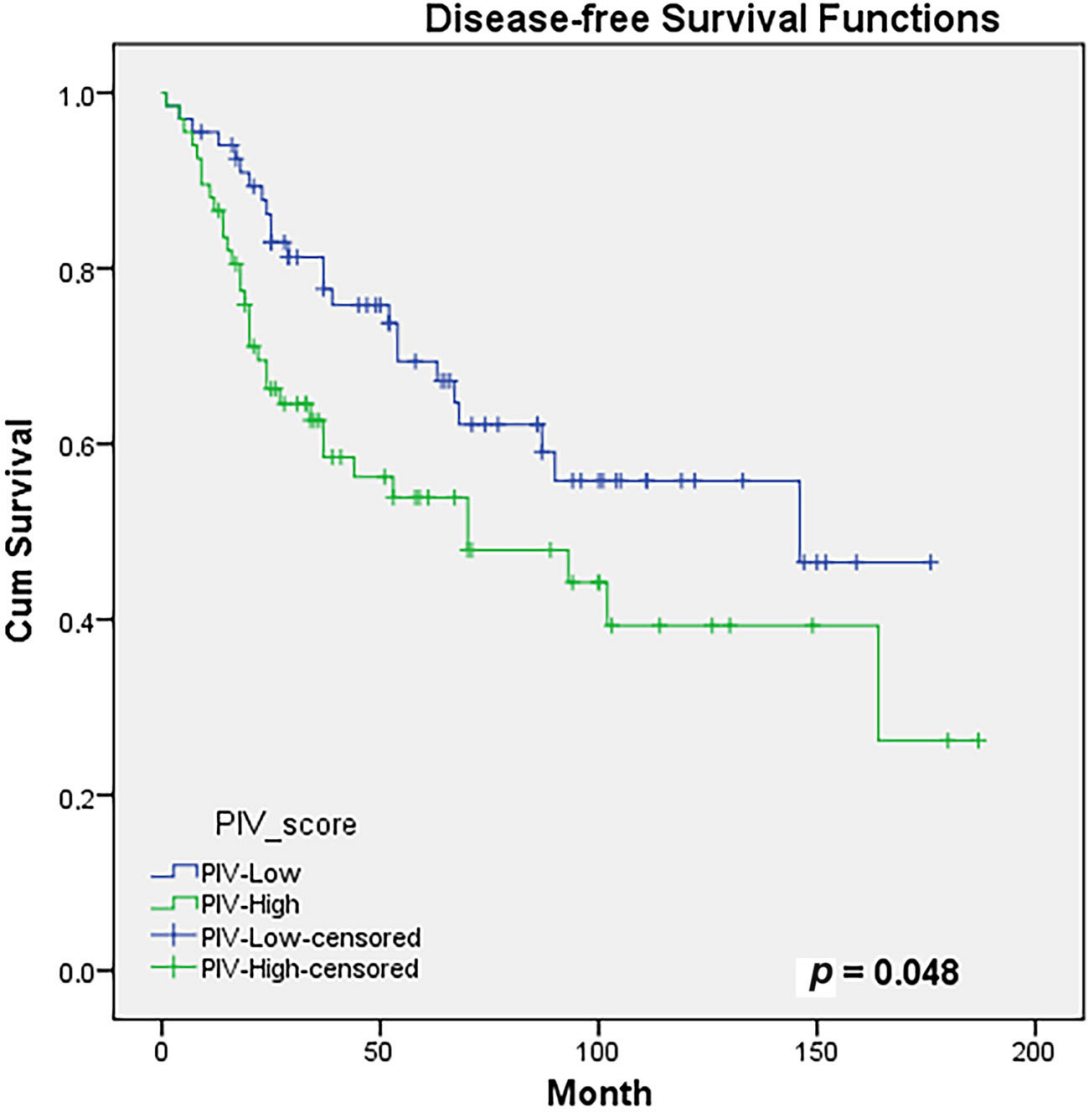
The Kaplan–Meier survival analysis with log rank test showing the disease‐free survival outcomes of groups according to PIV‐H and PIV‐L statuses: disease‐free survival curves of groups.

In the evaluation of the correlation between PIV score and DFS using the Kaplan–Meier test, the 5‐year DFS rates were 67% compared to 54%, while the 10‐year DFS rates were 56% versus 39% between the PIV‐L and PIV‐H groups, respectively (*p* = 0.048). Figure 2 shows the DFS curves according to the groups. In the evaluation of prognostic factors affecting DFS, the PIV score was found to be statistically significant in multivariate analysis (HR: 1.87, 95% CI: 1.08–3.26, *p* = 0.026) and insignificantly in univariate analysis (HR: 1.68, 95% CI: 0.99–2.86, *p* = 0.052). In addition to female sex, surgical margin positivity, and lymphovascular invasion were found statistically significant in both univariate and multivariate analyses. The prognostic factors affecting the DFS of PIV‐L and PIV‐H groups are given in Table 4.

**Table 4:** The prognostic factors affecting the overall survival of PIV‐L and PIV‐H groups.

Variables	Category	Univariate analysis		Multivariate analysis	
		HR (95% CI)	*p*	HR (95% CI)	*p*
PIV score	PIV‐L	RF		RF	
	PIV‐H	1.68 (0.99–2.86)	0.052	1.87 (1.08–3.26)	0.026
Sex	Male	RF		RF	
	Female	1.78 (1.06–3.01)	0.029	2.36 (1.35–4.12)	0.002
ECOG PS	ECOG 0	RF			
	ECOG 1	1.48 (0.84–2.60)	0.171		
	ECOG ≥ 2	2.12 (0.85–5.28)	0.105		
Localization	Proximal	RF			
	Middle	1.15 (0.39–3.38)	0.788		
	Distal	1.08 (0.38–3.06)	0.884		
Neoadjuvant treatment	CRT	RF	0.120		
	TNT	0.50 (0.21–1.19)			
Preoperative. N stage	Negative	RF			
	Positive	1.84 (0.98–3.44)	0.054		
Surgery type	LAR	RF			
	APR	1.56 (0.90–2.69)	0.107		
Pathological complete response	No	RF			
	Yes	0.51 (0.22–1.19)	0.123		
Tumor regression grade	Grade 0	RF			
	Grade 1	1.47 (0.52–4.15)	0.46		
	Grade 2	1.75 (0.69–4.46)	0.236		
	Grade 3	2.84 (1.15–7.03)	0.024		
Surgical margin	Negative	RF		RF	
	Positive	3.82 (2.03–7.22)	< 0.001	3.12 (1.59–6.13)	0.001
Lymphovascular invasion	No	RF		RF	
	Yes	2.71 (1.38–5.31)	0.004	2.22 (1.09–4.51)	0.027
Adjuvant therapy	No	RF			
	Yes	0.79 (0.45–1.37)	0.408		

Abbreviations: APR, abdominoperineal resection; CI, confidence interval; CRT, chemoradiotherapy; ECOG PS, Eastern Cooperative Oncology Group Performance Status; HR, hazard ratio; LAR, low anterior resection; PIV, pan‐immune‐inflammation value; TNT, total neoadjuvant treatment; RF, reference.

## 4 Discussion

The research indicated that patients with high PIV levels exhibited a higher occurrence of lymph node metastasis upon diagnosis, a lower likelihood of achieving complete pathological response postsurgery, a higher incidence of liver metastasis during follow‐up, elevated levels of CEA and CA19.9 tumor markers, a poorer tumor regression score, and a greater presence of tumor deposits and budding compared to patients with low PIV levels. Furthermore, patients with PIV‐H exhibited poorer outcomes in terms of OS and DFS. While PIV scoring was prognostic for OS in univariate analysis, this result lost its significance in multivariate analysis. For DFS, although a near‐significant result was obtained in univariate analysis, the PIV score was determined as an independent prognostic factor in multivariate analysis.

In cancer progression, inflammatory cells are crucial actors at all phases of the disease. Neutrophils and platelets play a role in the processes of epithelial–mesenchymal transition and angiogenesis. CD4+ T helper (Th) 2 and Th17 trigger metastasis and invasion, while CD8+, CD4+ Th1 T lymphocytes, and B lymphocytes have antitumoural effects. Monocytes process and present antigens, endorse humoral immunity, and pro‐inflammatory macrophages favor a TH1 response; however, they contribute to an immune‐suppressive tumor microenvironment [23]. Since the PIV score is calculated by considering four types of inflammatory cells, it can provide clinical information about the disease.

Zhao and colleagues researched colorectal cancer patients to analyze the connection between the patients' clinicopathological characteristics and baseline PIV scores [24]. In their study, the cutoff value for the PIV score was 159, which was determined by ROC analysis. The study demonstrated a strong association between baseline PIV score and tumor diameter, as well as CEA levels, while showing an inverse relationship with albumin levels. They showed that the T stage, N stage, and TNM stage were more advanced in patients with higher PIV scores. In a study by Sato and colleagues evaluating patients with preoperative stages I–III colorectal cancer, a high preoperative PIV score (> 375) was associated with T4 disease, poor tumor differentiation, and the presence of preoperative ileus. The study did not identify any association with lymph node metastasis [25]. Efil and colleagues found in their research on stages II–III colorectal cancer patients that PIV‐H status (median PIV > 490) was linked to right colon tumors, T4 tumors, tumor obstruction, and perforation [21]. The difference in PIV cutoff values resulted from the different methods used in the studies. Again, in a study of early‐stage colorectal patients operated on in two different countries, Park and colleagues showed that the systemic inflammatory responses of different races may vary [26]. In our study, the median PIV value was determined as 322. Our findings indicated that PIV‐H was associated with lymph node positivity at diagnosis, more advanced N stage after neoadjuvant therapy, and higher levels of CEA and CA19.9. Although the methods used to determine the PIV cut‐off value differed across studies, high PIV was generally associated with worse clinicopathological features. These findings indicate that PIV‐H is associated with a more advanced stage at diagnosis, unfavorable clinical features leading to increased disease‐related complications, aggressive behavior, and high‐risk disease. Balkwill et al. propose that the inflammatory cells and cytokines present in tumors predominantly promote tumor development, progression, and immunosuppression, rather than effectively triggering a host's antitumor defense [8]. Ding and colleagues identified direct links between IL‐17A, various serum metabolites, and the development of lung cancer, highlighting the significant impact of inflammatory and metabolic imbalances on the pathogenesis of cancer [27]. Thus, inflammatory biomarkers may be utilized to monitor the progression of cancer, and the severity of inflammation could be linked to the aggressive nature of the disease.

Current treatment guidelines recommend TNT, a new treatment algorithm, alongside the historical approach for the initial treatment of locally advanced rectal cancer [28]. TNT is particularly recommended for selected patients with high‐risk and poor clinicopathological features, including clinical T4 stage, extramural vascular invasion, clinical N2 disease, and the presence of metastatic lateral lymph nodes [28]. Also, mesorectal fascia and adipose tissue are important for the response to neoadjuvant treatment [29]. Along with previous research, inflammation markers like PIV‐H and others may serve as an additional factor in identifying high‐risk patients due to their correlation with unfavorable clinicopathological characteristics such as advanced T stage, N stage, and poor differentiation. This may be an easy‐to‐apply, noninvasive test that will influence the choice of treatment. Local recurrence in the course of rectal cancer is associated with a poor prognosis [30]. Neoadjuvant treatment has been brought to the agenda to reduce the risk of local recurrence in rectal cancer and to enable organ‐preserving surgery. Considering the effect of PIV score on neoadjuvant treatment response, in this study, pathological complete response and tumor response to treatment were found to be worse in the PIV‐H group. It was also noted that liver metastasis was higher during the follow‐up. Inflammation in the tumor microenvironment sustains carcinogenesis by contributing pro‐inflammatory cytokines, angiogenesis factors, and tumor‐associated immune‐tolerant macrophages. This immune modulation in the tumor microenvironment with all these factors allows the tumor to escape from the immune system [31, 32, 33, 34, 35]. This situation affects tumor behavior and the prognosis of the disease and has been linked to treatment resistance [35, 36]. In a meta‐analysis of studies on cancers involving immune checkpoint inhibitors, it was shown that the PIV value was associated with treatment response, and a high PIV value correlated with worse treatment outcomes [37]. Examining the tumor microenvironment requires tissue biopsy and complex pathological techniques, which can be invasive and sometimes impractical. It may be beneficial to investigate the tumor microenvironment by analyzing biomarkers in peripheral blood.

A meta‐analysis across different types of cancers revealed that assessing the PIV score before treatment can serve as a valuable indicator for predicting prognosis [38]. In a meta‐analysis by Hai‐Jing and colleagues, which included 30 trials, the prognostic significance of the PIV score on OS and progression‐free survival (PFS) was observed across various geographical regions, tumor stages, and treatment strategies [39]. Considering colorectal cancers, a meta‐analysis by Yang and colleagues indicated that patients in the high baseline PIV group had worse OS and PFS. Additionally, an early increase in PIV after treatment initiation was significantly associated with decreased OS and a trend toward PFS [40]. There is evidence that a high PIV score in metastatic disease may indicate a poor prognosis for OS and PFS [17, 41]. It has been noted that a rise in PIV score during treatment for metastatic disease could indicate disease progression [42]. A study on patients with metastatic colorectal cancer treated with immunotherapy revealed that high baseline PIV levels and an escalation in PIV scores during treatment were both significant independent indicators of OS and DFS [11]. In another study evaluating early‐stage colorectal cancer patients, PIV elevation was found to be associated with lower DFS and OS [21]. In our study, OS and DFS of patients with PIV‐H were found to be statistically significantly shorter than those with PIV‐L. This effect may have been influenced by the poorer clinicopathologic features of PIV‐H patients. Additionally, a poorer response to neoadjuvant treatment may have also contributed to this effect. In the present study, while PIV scoring was prognostic for OS in univariate analysis, this result lost its significance in multivariate analysis. For DFS, although a borderline significant result was obtained in univariate analysis, the PIV score was identified as an independent prognostic factor in multivariate analysis. While PIV scoring did not provide strong evidence for OS, its effect was more pronounced for DFS. In patients with rectal cancer, the pretreatment balance favoring excessive inflammation appears to negatively impact prognosis.

Since studies evaluating PIV scores in rectal cancer patients receiving neoadjuvant treatment are rare, this study is valuable. However, its retrospective nature and the limitation of being a single‐center study are notable shortcomings. In the literature, various methods (median, ROC analysis, R.) have been employed to determine the cut‐off point for the PIV value, resulting in different cut‐off values emerging.

## 5 Conclusion

This study demonstrated that pretreatment PIV score is important in terms of clinicopathology, treatment response, survival, and prognosis in patients with rectal cancer. The PIV score may be a biomarker that has the potential to be used routinely in treatment decision‐making and follow‐up of the disease since it is a noninvasive, easily accessible peripheral blood test that provides important data about the disease. Given the small number of participants included in the analysis, it is vital to interpret these results cautiously, indicating a clear need for larger sample sizes and multicentric, randomized controlled studies to further elucidate this issue.

## Author Contributions

**Mahmut Uçar:** conceptualization, methodology, writing – original draft. **Mukaddes Yılmaz:** data curation, validation, writing – original draft. **Eda Erdiş:** conceptualization, methodology, validation. **Birsen Yücel:** supervision, writing – review and editing.

## Ethics Statement

The present study was performed in line with the principles of the Declaration of Helsinki. Approval was granted by the Ethics Committee of Sivas Cumhuriyet University on November 16, 2023 (Permit # 2023‐11/12).

## Consent

Written consent was not necessary from participants due to the study's retrospective design and the protection of their anonymity.

## Conflicts of Interest

The authors declare no conflicts of interest.

## Data Availability

The data sets used and analyzed during the current study are available from the corresponding author upon reasonable request.
